# High Salt Intake Increases Copeptin but Salt Sensitivity Is Associated with Fluid Induced Reduction of Copeptin in Women

**DOI:** 10.1155/2014/641587

**Published:** 2014-10-23

**Authors:** Irina Tasevska, Sofia Enhörning, Philippe Burri, Olle Melander

**Affiliations:** ^1^Department of Internal Medicine, Skåne University Hospital, 20502 Malmö, Sweden; ^2^Department of Clinical Sciences, Lund University, 20502 Malmö, Sweden

## Abstract

This study investigated if copeptin is affected by high salt intake and whether any salt-induced changes in copeptin are related to the degree of salt sensitivity. The study was performed on 20 men and 19 women. In addition to meals containing 50 mmol NaCl daily, capsules containing 100 mmol NaCl and corresponding placebo capsules were administered during 4 weeks each, in random order. Measurements of 24 h blood pressure, body weight, 24 h urinary volume, and fasting plasma copeptin were performed at high and low salt consumption. Copeptin increased after a high compared to low dietary salt consumption in all subjects 3,59 ± 2,28 versus 3,12 ± 1,95 (*P* = 0,02). Copeptin correlated inversely with urinary volume, at both low (*r* = −0,42; *P* = 0,001) and high (*r* = −0,60; *P* < 0,001) salt consumption, as well as with the change in body weight (*r* = −0,53; *P* < 0,001). Systolic salt sensitivity was inversely correlated with salt-induced changes of copeptin, only in females (*r* = −0,58; *P* = 0,017). As suppression of copeptin on high versus low salt intake was associated with systolic salt sensitivity in women, our data suggest that high fluid intake and fluid retention may contribute to salt sensitivity.

## 1. Background

There is strong epidemiological support for a role of high salt intake in hypertension [1–5] and controlled interventions modulating salt intake have shown that high salt intake elevates blood pressure [6, 7]. The degree of blood pressure reduction following a lowering of salt intake (and the degree of blood pressure elevation following an increase of salt intake), that is, the degree of salt sensitivity, varies between individuals. The cause of the interindividual differences in salt sensitivity is unknown. In addition, it is unknown why salt elevates blood pressure. In particular, it is not known what role the increased water intake and water retention that commonly accompany a high salt intake have for salt-induced blood pressure elevation.

The two main stimulants of arginine vasopressin (AVP) secretion are hypovolemia and increased osmolarity. An increased salt intake is likely to lead to elevated AVP in order to retain water and thus sustain normal plasma osmolarity. At the same time, the expected increase in fluid intake and/or fluid retention following increased salt intake would be expected to lower AVP as a consequence of increased intravascular volume status and blood pressure. The net result of these two opposing effects of high salt intake on AVP is unclear. As we have previously found that high activity of the AVP system is related to components of the metabolic syndrome including both diabetes and hypertension [8–10] we here aimed to test whether a high salt intake would alter activity of the AVP system and, if that were the case, in which direction.

In addition, we hypothesized that the amount of water intake or water retention accompanying a standardized increase of salt intake may be reflected in changes of AVP secretion. If this were the case, the effect of water intake and/or retention on salt sensitivity could be estimated by measuring changes of AVP in plasma between low and high salt intake.

There are concerns regarding the reliability of AVP measurements in plasma, as AVP is an unstable molecule both* in vivo* and* ex vivo*, which requires complicated handling when sampling the patients' blood. Copeptin is a cleavage product of the C-terminal part of the AVP precursor hormone that is produced in equimolar amounts with AVP, a process similar to the generation of insulin and C-peptide. In contrast to AVP, copeptin is stable. Therefore, copeptin is found in considerably higher concentrations in plasma than in AVP and can be expected to be a more reliable marker of the true vasopressin release [11].

## 2. Methods

The study protocol has been described in detail previously [7]. Briefly, 46 unmedicated study subjects without history of hypertension, diabetes, or kidney disease were recruited via advertisements in local newspapers. Of these, 39 completed the study (20 men and 19 women). The mean age of the 39 subjects who completed the study was 53 ± 11 years and body mass index (BMI) was 26.3 ± 3.1. Clinical characteristics at baseline, low salt, and high salt are shown in Table 1. Subjects were first examined under baseline conditions (with subjects being on their habitual diets, i.e., on nonstandardized salt intake). During the entire study period of 8 weeks following the baseline visit, they were given all meals and drinks containing 50 mmol of NaCl (3 grams) and 50 mmol potassium per day. All meals and drinks were provided by our metabolic ward. The diet was designed by a dietician and daily energy intake was adjusted according to body weight and gender (2000–2600 kcal/day). All foods were produced and packed at Findus R&D AB (Bjuv, Sweden). Subjects were advised to consume everything they received from our ward/clinic but prohibited from ingesting anything else during the study apart from tap water. On top of the standardized diet each subject was given 100 mmol (6 grams) of NaCl per day (3 × 500 mg capsules 4 times daily) for 4 weeks and corresponding number of placebo capsules for 4 weeks in random order with double-blind crossover design. Thus, all subjects underwent two different 4-week periods with 150 mmol NaCl (9 grams) intake per 24 hours (high salt) and 50 mmol NaCl (3 grams) intake per 24 hours (low salt), respectively. Production of NaCl capsules and placebo capsules as well as blinding, coding, and packing procedures was performed by Apoteket AB (Swedish state pharmacy).

Subjects were examined with a similar protocol at baseline and at the end of the low salt and high salt periods. Venous blood was drawn and anthropometric measures were obtained and at the end of the visit an ABPM 90207 device (Spacelabs Medical Inc., Redmond, WA, USA) was applied on the left arm for 24-hour ambulatory blood pressure (ABP) measurement. Two different cuff sizes were used depending on the arm circumference (24–32 cm and 32–42 cm, resp.) of the study subjects. Study subjects were advised to maintain the left arm relaxed along the body during each measurement. During the 24 hours of ABP measurements, 24-hour urinary collections were also obtained. When subjects came to return the ABP device and the 24-hour urine collection any of the two different levels of salt intake started (or salt level changed). Degree of systolic and diastolic salt sensitivity was defined as the difference between 24-hour ABP after high salt and 24-hour ABP after low salt.

All study subjects had written informed consent and the study was approved by the Ethics Committee of Lund University.

### 2.1. Biochemical Assays

Urine and serum concentrations of sodium were measured by standard biochemical methods at the Department of Clinical Chemistry, Malmö University Hospital. Copeptin was measured in plasma using a commercially available assay in the chemiluminescence/coated tube format (B.R.A.H.M.S AG, Hennigsdorf, Germany) as described previously [12].

### 2.2. Statistics

All data were analyzed with SPSS statistical software (version 21, SPSS Inc., Chicago, IL, USA). Significance of differences of paired variables (i.e., changes induced by different levels of salt intake) was tested by paired *t*-test or Wilcoxon's paired rank test, where appropriate, whereas significance of differences between groups was tested with *t*-test. Pearson's test of correlations (*r*) was used to calculate correlations.

## 3. Results

The 24 h urinary excretion of sodium indicated a good compliance to the high and low salt diets (Table 1). The change of copeptin between the periods of low salt intake versus high salt intake is presented in Table 2. When increasing the dietary salt, copeptin increased significantly in all subjects, a result which was statistically significant in females but not in males. Increasing urinary volume correlated inversely with copeptin both at high and at low dietary salt intake in all individuals but not in females and males separately (Table 3). Furthermore, Table 4 shows salt-induced changes of body weight. Increasing body weight significantly correlated with a decrease in copeptin in all individuals when changing from high to low salt intake, whereas the change of urinary volume did not significantly correlate with any change in copeptin. When analyzing the change of ambulatory 24-hour blood pressure, systolic salt sensitivity correlated inversely with copeptin when going from low to high salt intake; however, this phenomenon was only seen in females (Table 5). On the other hand, systolic salt sensitivity was correlated neither with the change of body weight nor with the change of urinary excretion. Given the gender difference we divided the women in two categories, premenopausal and postmenopausal, to study whether estrogens contribute to the sensitivity of copeptin [13].  Table 6 shows salt-induced changes of copeptin in women aged below or above 51 [14]. A rise in copeptin with increasing dietary salt was only significant in women aged below 51 years. When comparing relating systolic salt sensitivity to salt-induced change of copeptin, there was no significant correlation in either female age group. Likewise, there was no relationship between salt sensitivity and salt-induced change of body weight and urinary volume in females below and above 51 years of age (Table 7).

## 4. Discussion

The main finding in our study is that even if copeptin increases after high salt intake, high salt-induced change of copeptin is inversely correlated with degree of salt sensitivity in females.

High level of copeptin in healthy subjects is associated with components of the metabolic syndrome [8, 9] including hypertension and independently predicts development of diabetes mellitus [10]. As it is an open question of whether high level of vasopressin (measured as copeptin) is causally related to diabetes, hypertension, and the metabolic syndrome or not, it is of interest to understand which environmental stimuli alter levels of copeptin. Such environmental stimuli can be used to test if subtraits of the metabolic syndrome can be ameliorated by reduction and worsened by stimulation of factors which elevate copeptin and thus provide information on causality. In the current study we selected the environmental factor of dietary salt, the effect of which on copeptin has never before been studied. We found salt of extra interest as it theoretically may have dual and opposing effects on AVP release by simultaneously increasing blood osmolality and blood volume. We found that 4 weeks of controlled high salt intake as compared to 4 weeks of controlled low salt intake increased plasma concentration of copeptin, suggesting that salt-induced increase of osmolality is a more potent stimulus for AVP secretion than salt-induced increase of blood volume would be as an inhibitor of AVP release. This suggests that if there were a causal relationship between copeptin and components of the metabolic syndrome, high salt intake might adversely affect such metabolic factors.

We found that copeptin was inversely related to urinary volume and that salt-induced increase of copeptin was inversely related to body weight gain when moving from low to high salt intake. The inverse relationship between copeptin and urinary volume was interpreted as being due to gradually increasing AVP suppression with increasing water intake and we thus assumed that low copeptin would be a surrogate marker for high water intake. Furthermore, we interpreted the inverse relationship between change of copeptin and change of body weight from low to high salt intake as gradually increased suppression of AVP as a result of increasing water retention.

As the role of high salt-induced water intake and water retention in salt sensitivity is controversial, we then tested whether reduction of copeptin in parallel with increased salt intake would be related to salt sensitivity. We found that among women, but not among men, this was in fact the case, suggesting that the salt sensitive component of blood pressure in women is in fact dependent on either a simultaneous high water intake or water retention or both of these factors. In summary, although increased salt intake in women on average leads to higher plasma concentration of copeptin, salt sensitivity in women is rather characterized by the opposite, that is, reduction of copeptin following a high salt intake presumably as a result of high water intake and water retention in those women. We thus believe that increased salt intake needs to be accompanied by increased intake and retention of water in order to lead to elevated blood pressure in women. In contrast, in men plasma concentration of copeptin is not affected by level of salt intake and there is no relationship between any salt-induced changes in copeptin and salt sensitivity of blood pressure, suggesting that different mechanisms underlie salt sensitivity in men.

### 4.1. Strengths and Limitations

The major limitation of our study is the relatively small number of subjects included, especially in the gender stratified analyses. Not to overestimate the magnitude of the demonstrated associations, our study needs replication. On the other hand, the salt sensitivity testing was performed under controlled conditions including double-blinded placebo versus salt capsule administration and random order of the high and low salt periods. In addition, 24-hour ABP was used to measure blood pressure, resulting in a more exact blood pressure phenotype compared to office blood pressure measurements.

## 5. Conclusion

The increase in dietary salt consumption significantly correlates with the increase in levels of copeptin in all individuals when calculated together and in females but not in males when calculated separately. An inverted correlation is observed comparing the urinary volume (being an indirect measure of fluid intake) with the levels of copeptin during low and high dietary salt consumption. Comparing the change in body weight (an indirect measure of fluid retention) with the change in levels of copeptin from low dietary salt intake to high salt intake, we can establish a negative correlation being significant in all individuals. Our findings suggest that copeptin can be a useful marker for the amount of fluid consumption as well as fluid retention. Due to the gender differences concerning the sensitivity of copeptin release during low and high dietary salt consumption we also hypothesize that estrogens are contributing factors to the degree of this sensitivity.

## Figures and Tables

**Table 1:** Clinical characteristics of study subjects.

	Baseline	High salt	Low salt	*P* ^*^ (high versus low salt)
24-hour SBP (mmHg)	139 ± 13,3	136 ± 12,7	131 ± 11,1	<0,0001
24-hour DBP (mmHg)	86,3 ± 7,4	85,0 ± 7,0	82,3 ± 6,6	0,004
Body weight (kg)	79,5 ± 11,2	77,4 ± 10,7	77,3 ± 10,6	0,43
Urine-Na^+^ (mmol/24 h)	165 ± 67,4	140 ± 39,5	50,7 ± 17,3	<0,0001
Serum-Na^+^ (mmol/L)	140 ± 1,8	141 ± 1,5	139 ± 1,7	<0,0001

^*^Refer  to individual change of variable on high salt as compared with that on low salt (i.e., variable Δ-value). SBP, systolic blood pressure; DBP, diastolic blood pressure.

**Table 2:** Changes in copeptin during low salt consumption and high salt consumption and the changes in between.

	Copeptin low salt	Copeptin high salt	ΔCopeptin	*P*
All	3,12 ± 1,95	3,59 ± 2,28	0,47 ± 1,14	0,02
Female	2,19 ± 1,38	2,75 ± 1,64	0,56 ± 0,83	0,02
Male	3,89 ± 2,04	4,29 ± 2,53	0,40 ± 1,37	0,22

ΔCopeptin, the change of copeptin during high salt intake and low salt intake; *P*, significance.

**(a):** 

Copeptin low salt		
	*r*	*P*
Urine volume: all	−0,42	0,01
Urine volume: female	−0,51	0,05
Urine volume: male	−0,39	0,09

*P*, significance.

**(b):** 

Copeptin high salt		
	*r*	*P*
Urine volume: all	−0,60	0,001
Urine volume: female	−0,36	0,16
Urine volume: male	−0,65	0,003

*P*, significance.

**Table 4:** Correlations between the change in copeptin and change in weight as well as change in 24 h urinary volumes, during low and high salt consumption.

ΔCopeptin						
	All		Female		Male	
	*r*	*P*	*r*	*P*	*r*	*P*
ΔWeight	−0,53	<0,001	−0,54	0,03	−0,54	0,02
ΔUrine volume	0,14	0,42	0,24	0,36	0,06	0,80

ΔCopeptin, the change of copeptin during high salt intake and low salt intake; ΔWeight, the change in weight; ΔSBP, systolic salt sensitivity; ΔUrine volume, the change in urinary volume from high to low urinary volume; *P*, significance.

**Table 5:** Correlations between the change in systolic salt sensitivity and change in copeptin, in weight, and in 24 h urinary volumes, during low and high salt consumption.

ΔSBP						
	All		Female		Male	
	*r*	*P*	*r*	*P*	*r*	*P*
ΔCopeptin	−0,21	0,22	−0,58	0,02	−0,05	0,83
ΔWeight	0,27	0,08	0,33	0,18	0,25	0,29
ΔUrine volume	0,05	0,78	−0,13	0,61	0,27	0,25

ΔSBP, systolic salt sensitivity; ΔCopeptin, the change of copeptin during high salt intake and low salt intake; ΔWeight, change in weight; ΔUrine volume, change in urinary volume from high to low urinary volume; *P*, significance.

**Table 6:** Comparison between pre- and postmenopausal women of changes in copeptin during low salt consumption and high salt consumption and the change in between.

	*N*	Copeptin low salt	Copeptin high salt	ΔCopeptin	*P*
Female < 52 yrs	7	2,09 ± 1,84	3,14 ± 2,40	1,05 ± 0,74	0,01
Female ≥ 52 yrs	9	2,27 ± 1,02	2,45 ± 0,71	0,18 ± 0,71	0,48

ΔCopeptin, the change of copeptin during high salt intake and low salt intake; *N*, number of participants in each category; *P*, significance.

**Table 7:** Change in systolic salt sensitivity and the change in copeptin, in weight, and in 24 h urinary volumes, during low and high salt consumption in pre- and postmenopausal women.

ΔSBP				
	Female < 51 yrs *N* = 7		Female ≥ 51 yrs *N* = 9	
	*r*	*P*	*r*	*P*
ΔCopeptin	−0,60	0,16	−0,41	0,27
ΔWeight	0,49	0,22	−0,24	0,49
ΔUrine volume	−0,19	0,65	−0,04	0,90

ΔSBP, systolic salt sensitivity; ΔCopeptin, the change of copeptin during high salt intake and low salt intake; ΔWeight, change in weight; ΔUrine volume, change in urinary volume from high to low urinary volume; *N*, number of participants in each category; *P*, significance.
